# MicroRNA Bta-miR-24-3p Suppressed Galectin-9 Expression through TLR4/NF-ĸB Signaling Pathway in LPS-Stimulated Bovine Endometrial Epithelial Cells

**DOI:** 10.3390/cells10123299

**Published:** 2021-11-25

**Authors:** Ayodele Olaolu Oladejo, Yajuan Li, Wenxiang Shen, Bereket Habte Imam, Xiaohu Wu, Jie Yang, Xiaoyu Ma, Yanan Lv, Wei Jiang, Xuezhi Ding, Shengyi Wang, Zuoting Yan

**Affiliations:** 1 Key Laboratory of Veterinary Pharmaceutical Development of Ministry of Agriculture, Lanzhou Institute of Husbandry and Pharmaceutical Sciences of Chinese Academy of Agricultural Science, Lanzhou 730050, China; oladejoayodele85@gmail.com (A.O.O.); 18719826933@163.com (Y.L.); shane095@foxmail.com (W.S.); Bekihi14@gmail.com (B.H.I.); wuxiaohu01@caas.cn (X.W.); 15535384771@163.com (J.Y.); 18903865563@163.com (X.M.); lvyanan01@caas.cn (Y.L.); gw18848970753@126.com (W.J.); dingxuezhi@caas.cn (X.D.); 2 Department of Animal Health Technology, Oyo State College of Agriculture and Technology, Igboora 201103, Nigeria; 3 Department of Veterinary Science, Hamelmalo Agricultural College, Keren 397, Eritrea

**Keywords:** endometritis, Bta-miR-24-3p, LGALS9, NF-ĸB, TLR4, endometrial cells

## Abstract

Endometritis is a major infectious disease affecting dairy development. MicroRNAs are recognized as critical regulators of the innate immune response. However, the role and mechanism of Bta-miR-24-3p in the development of endometritis are still unclear. This study aimed to investigate the effect of Bta-miR-24-3p on the inflammatory response triggered by lipopolysaccharide (LPS) and to clarify the possible mechanism. LPS-treated bovine endometrial epithelial cells (BEECs) were cultured to investigate the role of Bta-miR-24-3p. The expression levels of Bta-miR-24-3p were downregulated, and galectin-9 (LGALS9) were measured by quantitative real-time polymerase chain reaction. The LPS-induced inflammatory response was assessed by the elevated secretion of inflammatory cytokines measured by using enzyme-linked immunosorbent assay and quantitative real-time polymerase chain reaction. Activation of nuclear factor-κB (NF-κB) and TLR4 pathway was assessed by Western blot. The interaction between Bta-miR-24-3p and LGALS9 was validated by bioinformatics analysis and a luciferase reporter assay. LPS-induction in BEECs with Bta-miR-24-3p was overexpressed leads inhibition of pro-inflammatory cytokines, LGALS9 expression, and TLR4/NF-ĸB pathway deactivation. Knockdown of LGALS9 inhibited the LPS-induced inflammatory response in BEECs. LGALS9 was validated as a target of Bta-miR-24-3p. Cloned overexpression of LGALS9 failed to alter the effect of Bta-miR-24-3p on the inflammatory response in BEECs. Overall, Bta-miR-24-3p attenuated the LPS-induced inflammatory response via targeting LGALS9. The immunotherapeutic stabilisation of Bta-miR-24-3p could give a therapeutic option for endometritis and other disorders commonly associated with endometritis, suggesting a novel avenue for endometritis treatment.

## 1. Introduction

Endometritis is an infection of the postpartum uterus common in postpartum dairy cows. It is linked to poor reproductive performance, as evidenced by lower first-service conception rates, a lower risk of pregnancy across the breeding cycle, and a higher risk of reproductive culling [1,2]. After resolution of clinical condition without treatment, endometritis lengthens the interval between parturition and the first insemination and delays conception, resulting in higher culling rates for reproductive insufficiency [3,4,5]. Many pregnancy failures in natural and assisted reproductive technology pregnancies can be linked to insufficient endometrial receptivity, which is described as the physiological condition of the uterus when fertilized embryo growth and implantation are possible for the maintenance of pregnancy [6]. Bacteria often cause endometritis and reduce fertility in dairy cattle. The presence of subclinical endometritis in a higher incidence of repeated breeder cows (RBC) is the highest known risk factor for the infections, leading to a delayed resumption of postpartum ovarian cycles and insemination failure [5,7,8]. Previous studies have demonstrated that a cellular immune response in the endometrium, occurring during subclinical endometritis, might play a role in subfertility/infertility in RBC [9,10]. There are normal physiological needs to provide the endometrium-derived trophic factors toward the early developing embryo floating in the endometrial lumen [11], but this was usually hindered due to endometrial infection progression. Several endometrial genes have been incriminated in the pathophysiology of endometritis, which depicts the presence of gene transcription, cell skeleton, cell proliferation, cell apoptosis, and signal transduction to immune function, inflammation, infection, and disease [12,13,14,15,16]. Galectin families are essential for establishing an immune-privileged local milieu for implantation and early fetal development, related to immunosuppressive actions and crucial in maternal-fetal tolerance in humans and rodents [17,18,19,20]. This function has not been established in the cow. Galectin-9 (LGALS9) is expressed in all uterine cell types, including endometrial epithelial and endothelial cells [15,18,21]. LGALS9 is involved in cell development, attachment processes, innate and adaptive immunity regulation, inflammatory response, and immunosuppressive activity regulation [15,22]. In bovine epithelial endometrial cells, the in vitro effects of LPS treatment on gene expression patterns were investigated, and gene expression of LGALS9, which encodes proteins critical for early pregnancy, was shown to be dramatically overexpressed [12,13,23,24]. In addition, over-expression of LGALS9 associated with the increased expression of many genes coding pro-inflammatory cytokines suggests that LGAL9 may control these molecules’ production in the absence of immune cells [25,26,27]. Lipopolysaccharide (LPS), a major component of cell walls unique to most gram-negative bacteria, stimulates immune system cells and induces a strong inflammatory response [13,15]. LPS expressed on the Gram-negative bacteria surface usually enhances the activation of pro-inflammatory pathways, deregulates the function of endometrial cells, and plays a key role in the mechanisms involved in endometritis [17]. LPS mediated its penetrating destruction of the endometrial cell walls through the pathogens recognizing receptors (PRR), and the most studied PRR in the molecular and cellular pathogenesis cascade of endometritis is the Toll-like receptor 4 (TLR4) [28]. TLRs are widely known for their importance in the innate immune response to microbial invasion. TLR4 dependent innate immune signaling systems are required for bovine endometrial epithelial cells to respond to LPS stimulation [15,29]. NF-κB, a critical nuclear transcription factor, has been linked to the pathogenesis of endometritis and shown to regulate the generation of pro-inflammatory mediators [28,30]. Endometrial cells release inflammatory cytokines and their receptors (TLR4), crucial in controlling fetal development and interactions with maternal cells, especially during implantation [31]. Tumor Necrosis Factor-alpha (TNF-α) has pleiotropic effects on cell growth, inflammation, and innate immunity in the endometrium and is strongly involved in embryo development and implantation [32,33]. TLR4 signaling pathway leads to the generation of numerous pro-inflammatory cytokines such as TNF-α, IL-1β, and IL-6 and chemokine such as IL-8 associated with inflammatory reactions [34,35,36].

MicroRNAs (miRNAs) are endogenous single-stranded non-coding RNA with 18–25 nucleotides in length. MiRNA controls the gene expression at the post-transcriptional level by inducing RNA degradation or limiting mRNA translation. MiRNA levels in the cell and extracellular vesicles have been associated with many diseases, including inflammation and cancer [37,38,39]. Recent research evidence reveals that miRNAs have a role in different pathophysiological processes of diseases, including cell development, death, signal transduction, and degenerative pathologies [40]. MiRNA attaches to the target gene’s 3′UTR seed sequence to trigger deterioration of the target mRNA, therefore partaking in various biological processes such as cell proliferation, death, and differentiation [41,42]. Several microRNAs have been reported to exert inflammation suppressive potentials against endometritis [13,15,41,43]; hence with the abundance of miR-24 in the endometrial epithelial cell; there is no evidence of research evaluation of it on the pathogenesis, and the regulation of endometrial inflammation incriminating gene like LGALS9; called for concerns to investigate the role of Bta-miR-24-3pin the pathogenesis of bovine endometritis and its gene regulatory functions.

We hypothesized that Bta-miR-24-3p has an immunomodulatory effect on LPS-induced endometritis through TLR4/NF-ĸB signaling pathway targeting LGALS9. It was also hypothesized that Bta-miR24-3p might act as a potent inflammatory regulator and a potential target for treating inflammatory diseases like endometritis.

## 2. Materials and Methods

### 2.1. Reagent and Chemical

The reagents and chemicals used for the research are ELISA Kit for IL-1β, 1L-6, IL-8, and TNFα, Precast SDS gel, PBS, TBST, (Solarbio, Beijing, China), DAPI with anti-fade mount (Beyotimes Biotech, Shanghai, China), EZNA^®^ Endo-Free Plasmid DNA Midi kit (Omega Bio-Tek, Norcross, GA, USA), Trypsin-EDTA(Gibco, Big Cabin, OK, USA), Trizol reagent(ThermoFisher, Waltham, MA, USA), Evo M-MLV RT Kit and SYBR Green Premix Pro Taq HS qPCR kit (Accurate Biotech, Shanghai, China). 10% foetal bovine serum, DMEM/F12 dilution (HyClone, Logan, UT, USA (Anti-p65 (ab1932238, Abcam, London, UK), GP transfer mate (GenePharma, Shanghai, China), Lipopolysaccharide (Escherichia coli 0111:B4) from Sigma-Aldrich, Saint Louis, MO, USA. Lipofectamine^TM^ 3000 and 2000 (Invitrogen, Austin, TX, USA).

### 2.2. CellIsolation, Culture, and Preservation

The healthy uteri from Ten Chinese Holstein dairy cows were used to get BEECs. Briefly, a healthy uterus was brought to the laboratory in a sterile PBS (pH 7.2) containing penicillin (100 μg/mL) and streptomycin (100 U/mL). Endometrium from the uterine horn was cut off into 2–3 cm long pieces, washed in PBS (pH 7.2) twice. Then uterine tissue was digested with 1% collagenase I (Sigma, St. Louis, MO, USA) diluted in DMEM/F12 (HyClone, Logan, UT, USA) for 6 h. The digested endometrium was scraped using a sterile cell scraper, and scraped materials were collected and washed in PBS (pH 7.2). Then, the collected materials were centrifuged at 100 g for 5 min to collect the cell suspension. Trypan Blue stain was used to estimate cell viability. Cells were cultured in DMEM/F12 with 10% fetal bovine serum (Gibco, Big Cabin, OK, USA), penicillin (100 IU/mL), and streptomycin (100 μg/mL) at 37 °C with 5% CO_2_ and 95% sterile air when the viability >95%. The medium was changed every 3 days until the cells reached approximately 90% confluence. The cultures were inevitably mixed with some stromal cells; stromal cells were removed according to the different sensitivities of epithelial cells and stromal cells to Trypsin (Gibco, Big Cabin, OK, USA), [16]. Time-different digestion was conducted thrice to obtain purified epithelial cells, and the cells are now a pool with no differentiation cow specificity. The picture of bovine endometrial epithelial cells was presented in Supplementary Figures S1–S3. The cell viability was examined by the Trypan Blue stain method. Subsequently, cells were cultured in DMEM/F12 with 10% fetal bovine serum at 37 °C with 5% CO_2_ and 95% sterile air when the viability >95%. The medium was changed within a 48 h interval until the cells reached approximately 90% confluence, and cells were cryopreserved as the pool at −80 °C before usage. The cell density of 2 × 10^5^ cells /well was transferred into the 6-well plate for 24 h; the supernatant was then replaced with new DMEM containing different concentrations of LPS and 10% FBS and cultured for an additional 24 h.

### 2.3. Transfection of Bovine Endometrial Epithelial Cell with miRNA and si-LGALS9

Overexpression and knockdown of miRNA and LGALS9 respectively were achieved by transfection of miRNA mimic or miRNA inhibitor and their negative control (NC) and siLGALS9 and si-NC into BEECs. Cells used for cutting were cryopreserved, passaged twice, cultured, and seeded at a density of 2 × 10^5^ cells/well. The synthetic Bta-miR-24-3p mimic, Bta-miR-24-3p inhibitor, negative control RNA (control mimic and control inhibitor), siLGALS9, and si-NC were purchased from GenePharma (Shanghai, China). BEECs were seeded in 6-well plates using 10% FBS in DMEM. Once the cells were 70–80% confluent, the medium was changed to DMEM Reduced Serum Medium (Hyclone, USA) before transfection. Then, equal amounts of Bta-miR-24-3p mimic, negative control mimic, Bta-miR-24-3p inhibitor, negative control inhibitor, si-LGALS9, and si-NC were transfected into the cells in different plates using GP-Transfect-Mate^®^ (GenePharma, Shanghai, China) according to the manufacturer’s instructions. After 24–48 h, the cells were harvested, and the expression of LGALS9 and Bta-miR-24-3p respectively was detected by western blotting and quantitative real-time polymerase chain reaction (qRT-PCR). The sequences of the RNA oligonucleotides are listed in Supplementary Table S1.

### 2.4. Cell Counting Kit-8 (CCK-8) Assay

After treatment with the required experimental plan, cell viability was examined using a cell counting kit-8 (CCK-8) assay kit (Beyotimes, Shanghai, China). The BEECs (5 × 10^4^ cells/well) from cryopreserved samples randomly, after 2–3 passages were incubated at 37 °C for 1 h in 96-wells plates and; the cells were treated with 3 µg/mL of LPS for 0, 6, 12, 24, and 36 as well as the pcDNA 3.1 with each group having five replicates. After the cells were incubated with 10 µL of CCK-8 solution for 4 h at 37 °C, the microplate reader was used to read the absorbance at 450 nm (Bio-Rad Instruments, Hercules, CA, USA).

### 2.5. RNA Extraction, cDNA Synthesis, and Reverse Transcription Quantitative Polymerase Chain Reaction 

Isolation of total RNA of BEECs from the transfected and designated experiment was done with TRIzol reagent (Invitrogen, Austin, TX, USA). The OD values of the RNA were analysed with a Q5000 and evaluated for concentration and purification at 260 and 280 nm and the ratio of OD260 to OD280 of all samples. Genomic DNA contamination of all RNA samples was removed with an RNase-free DNase. The RNA was transcribed into cDNA using the Emo-M-MLV RT kits (Accurate Biotech, Changsha, China) according to the manufacturer’s instructions. The customized kit and primers of Bta-miR-24-3p and U6 small nuclear RNA (snRNA) were purchased from Gene Pharma, Shanghai, China. RT-qPCR was performed using customized miRNA real-time PCR kits (Gene Pharma, Shanghai, China), and mRNA analysis was performed using SYBR^®^ Green Premix Pro Taq H.S. kits (Accurate Biotech, Changsha, China). The primer sequences for the inflammatory cytokines and LGALS9 for RT-qPCR are shown in Supplementary Table S2. The relative expression levels of Bta-miR-24-3p and mRNA were normalized to the endogenous reference U6 and β-actin, respectively, according to the 2^−ΔΔCt^ method.

### 2.6. Extraction of Protein and Western Blot Analysis

Experimental and transfected cells were collected, homogenized, and lysed using TPEB^®^ assay reagent (Bio Sharp, Wuhan, China), containing 0.5 mM of phenylmethanesulfonyl. Protein concentration was determined by BCA protein assay (Takara, Shanghai, China). Samples of 25 μg protein were fractionated by SDS-PAGE in 10% gradient Tris-glycine precast gels (Solarbio, Shanghai, China) and transferred to methanol activated-polyvinylidene difluoride (PVDF) membrane (Millipore, Billerica, MA, USA). The membrane was incubated for 1 h in a blocking solution of QuickBlock^TM^ Western (Beyotimes Biotech, Wuhan, China) for 15–20 min at room temperature with gentle shaking at 4 °C with overnight incubation in primary antibodies against p65 (1:1000; Proteintech, Wuhan, China), p-p65, IkBα, p-IkBα, and β-actin (1:500, 1:800, 1:500, 1:600 and 1:1000 dilution rates respectively; Abcam, London, UK), TLR4, (1:500, Proteintech, China), IRAK4, MyD88 and TRAF6 (1:400, 1:300:1:500, Beyotimes Biotech, Shanghai, China). Subsequently, the labelled proteins were visualized by incubation with a horseradish-peroxidase (HRP) conjugated anti-rabbit IgG (1:50,000; Abcam, London, UK) followed by development with a chemiluminescence substrate (ECL) for HRP (Thermo Fisher Scientific, San Diego, CA, USA). The images of western blots were captured by GE ImageQuant.2.11.

### 2.7. Enzyme-Linked Immunosorbent Assay (ELISA) 

After the designated treatment, the secretion levels of tumor necrosis factor-α (TNF-α), interleukin-1β (IL-1β), IL-8, and IL-6 in cell supernatants were measured using particular commercial enzyme-linked immunosorbent assay (ELISA) kits (Solarbio, Beijing, China) following the manufacturer’s instructions. ELISA results were detected using a microplate reader (BioTek’s Epoch™, Winooski, VT, USA) and analysed according to the standard curve.

### 2.8. Immunofluorescence Techniques

The BEECs (2 × 10^5^ cells/well) transfected and treated was used to perform immunofluorescence staining. Briefly, cells were fixed with 4% paraformaldehyde for 20 min; then, they were immersed with PBS in 1% Triton X-100 to permeabilize the cells at room temperature for 20 min. The PBS washing of the permeabilized cells was repeated thrice, cleaning and blotting with filter paper and blocking for 20 min with 10% goat serum at room temperature. Each cell well in plates with a specific primary antibody (1:200) was incubated overnight at 4 °C and then incubated with FITC-labeled secondary antibodies (1:400) in the dark box for 1 h at 37 °C. Finally, the nuclei were stained using DAPI (Abcam, Shanghai, China) for 10 min, washed with PBS, sealed with an anti-fluorescence quencher, and captured fluorescent images with an Operetta high-content molecular Image Screening System Fluorescence Microscope (PerkinElmer, Inc., Chicago, IL, USA). The IOD and area of cells were measured by Image-Pro Plus software, and the fluorescence intensity was expressed as IOD/area.

### 2.9. Target Prediction of Bta-miR-24-3p and Luciferase Reporter Assay

TargetScan, miRanda, and picTar prediction software were used to predict the target genes of Bta-miR-24-3p based on the seed region of Bta-miR-24-3p and 3′-UTR of LGALS9. According to the prediction, LGALS9 was found to be one of the targets of Bta-miR-24-3p. The corresponding MUT fragment of LGALS9 (MUT) and the WT fragment of LGALS9 (LGALS9-WT) containing potential binding sites was inserted into the pmirGLO-reporter plasmid Vector (GenePharma, Shanghai, China) to produce an LGALS9 luciferase reporter gene construct. According to the manufacturer’s instructions, the vector and Bta-miR-24-3p mimic were co-transfected into HEK293T cells using Lipofectamine 2000 reagent (Invitrogen). HEK293T cells were cultured in Dulbecco’s modified Eagle’s medium (DMEM) supplemented with 10% fetal bovine serum (FBS) at 37 °C in a humidified atmosphere containing 5% CO_2_. According to the manufacturer’s instructions, the pmiRGLOvector and Bta-miR-24-3p mimic were co-transfected into well cultured HEK293T cells using GP-transfect Mate reagent (GenePharma, Shanghai, China). After 24 h of transfection, the cells were detected with a luciferase detection kit and the Renilla luciferase/ Firefly luciferase assay kit.

### 2.10. Recombinant Plasmid Construction and Transfection

The recombinant plasmid pcDNA3.1(+)LGALS9 was digested by KpnI and XhoI at 37 °C overnight. The LGALS9-encoding gene was segregated, reclaimed, and depurated. Then the depurated products were ligated with pcDNA3.1(+) by 5μLLGALS9 DNA ligase (GeneCreate, Wuhan, China) at a ratio of 4:1 at 4 °C overnight. The bacterial *E. coli* X-L1-Blue transformed with pcDNA3.1(+)LGALS9 was cultured in L.B. medium with amino-benzyl penicillin-based overnight at 37 °C. The constructed cloned plasmid DNA was schematically represented in Supplementary Figure S4. The pcDNA3.1(+)LGALS9 plasmid was extracted with the Endo-Free Plasmid DNA Midi kit (Omega Bio-Tek, Norcross, GA, USA) based on the manufacturer’s instructions digested by KpnI and XhoI at 37 °C for 3 h. Finally, the products were separated, extracted, and purified. The cryopreserved BEECs were thawed, passage 3 times, and cultured for 24 h to 70–90% confluent (1 × 10^6^ cells/well). Transfection was carried out in a 6-wells plate according to the instructions for the Lipofectamine^TM^ 3000 (Invitrogen, USA) as the transfection reagent. 5 μg of pcDNA3.1(+)LGALS9, 5 μg of pcDNA3.1(+)LGALS9 with 50 nM Bta-miR-24-3p mimic, pcDNA3.1empty vector, pcDNA3.1empty vector and LPS, and pcDNA3.1empty vector with 50 nM Bta-miR-24-3p mimic were transfected separately into BEECs. The efficiency of transfection was examined by RT-qPCR and Western Blot techniques. The supernatant was collected to evaluate the concentration of the pro-inflammatory cytokines.

### 2.11. Data Statistics

All data collected in the experiment were performed with GraphPad Prism^®^ 7 and 9 (GraphPad, San Diego, CA, USA). All experimental results are displayed as the mean ± SEM, with at least three independent replications. The statistically significant difference between groups was tested by student *t*-test and One-way ANOVA (Analysis of Variance) as the data is required. We considered * *p* < 0.05; ** *p* < 0.01 to be statistically significant. 

## 3. Results

### 3.1. Reduced Expression of Bta-miR-24-3p and Elevation of LGALS9 following LPS-Stimulation In Vitro

**Figure 1:**
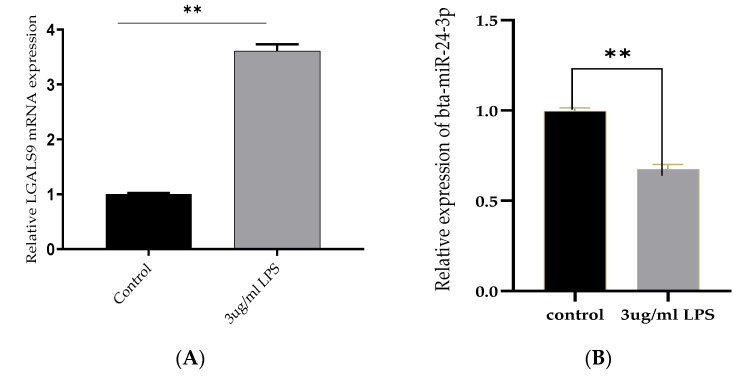

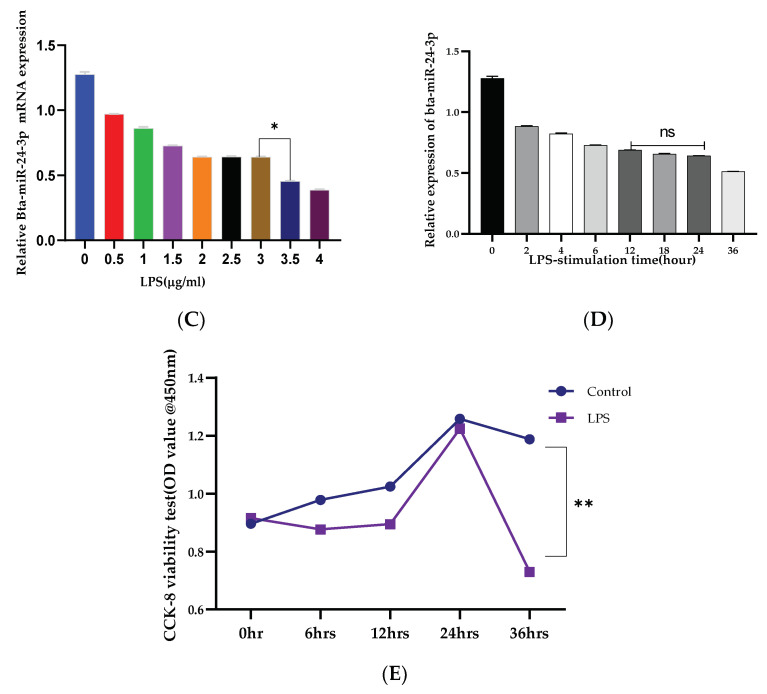
Expression of Bta-miR-24-3p and Galectin 9 gene(LGALS9) in BEECs stimulation with LPS. (**A**) BEECs were triggered with 3 μg/mL LPS concentrations for 24 h, and RT-qPCR measured the expression of LGALS9. (**B**) The BEECs were stimulated with 3 µg/mL LPS for periods of 24 h, and RT-qPCR measured the Bta-miR-24-3p expression level. The relative expression of Bta-miR-24-3p was normalized to U6 snRNA. (**C**) Different concentrations of LPS were used to induce BEECs for 24 h, revealing the different levels of Bta-miR-24-3p expression. (**D**) The BEECs were stimulated with 3 µg/mL LPS for additional periods, and RT-qPCR measured the Bta-miR-24-3p expression level. The relative expression of Bta-miR-24-3p was normalized to U6 snRNA. (**E**) The cell viability was investigated within different periods after treatment with 3 μg/mL LPS using the CCK-8 kit. The results are presented as the average of three experimental observations with mean ± SEM. * *p* < 0.05; ** *p* < 0.01. (Student *t*-test or One-way ANOVA).

The inflammatory model was established by BEECs stimulation with LPS for 24 h, and the mRNA expression level of Bta-miR-24-3p and LGALS9 genes was evaluated. The mRNA expression level of LGALS9 was significantly upregulated (*p* < 0.01). (Figure 1A), while a significant decrease (*p* < 0.01) in the expression of Bta-miR-24-3p was observed, as shown in Figure 1B. BEECs were stimulated with different concentrations of LPS and induced by 3 μg/mL of LPS at different times. The stimulation response showed that Bta-miR-24-3p exhibited a dose- and time-dependent down-regulation (Figure 1C,D). In addition, a CCK-8 assay was performed to investigate whether cell viability was affected by LPS administration and to determine the optimal timing for stimulation. The results showed that LPS (3 μg/mL) did not affect cell viability (Figure 1E), indicating that LPS stimulation did not significantly affect cell growth and proliferation. LGALS9 expression was significantly increased, and Bta-miR-24-3p was significantly downregulated; therefore, it affirmed that both LGALS9 gene and Bta-miR-24-3p are incriminated immune-inflammatory responses during endometritis.

### 3.2. Inhibiting the Expression of Pro-Inflammatory Cytokines and Restrains Activation of the TLR4/NF-κB Pathway by Bta-miR-24-3p in BEECs

**Figure 2:**
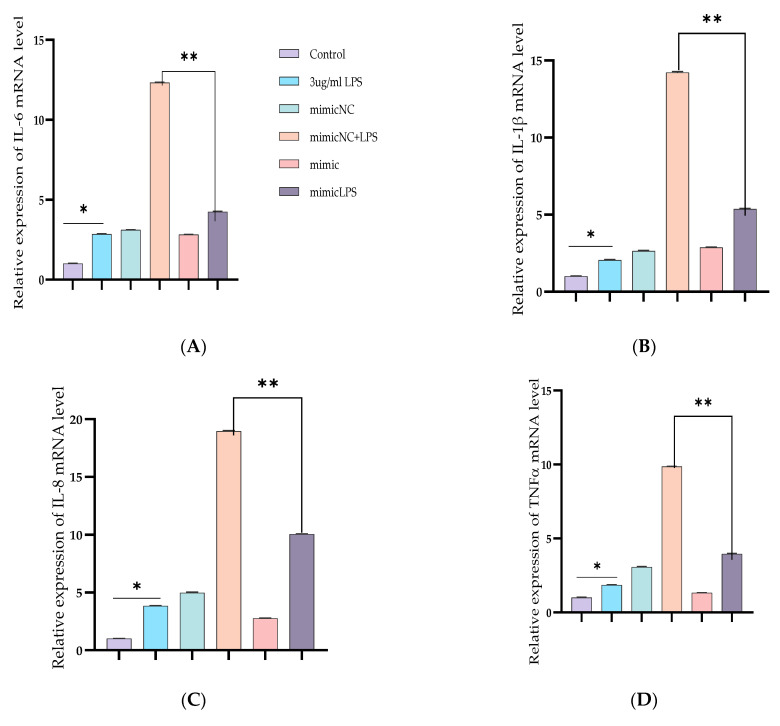

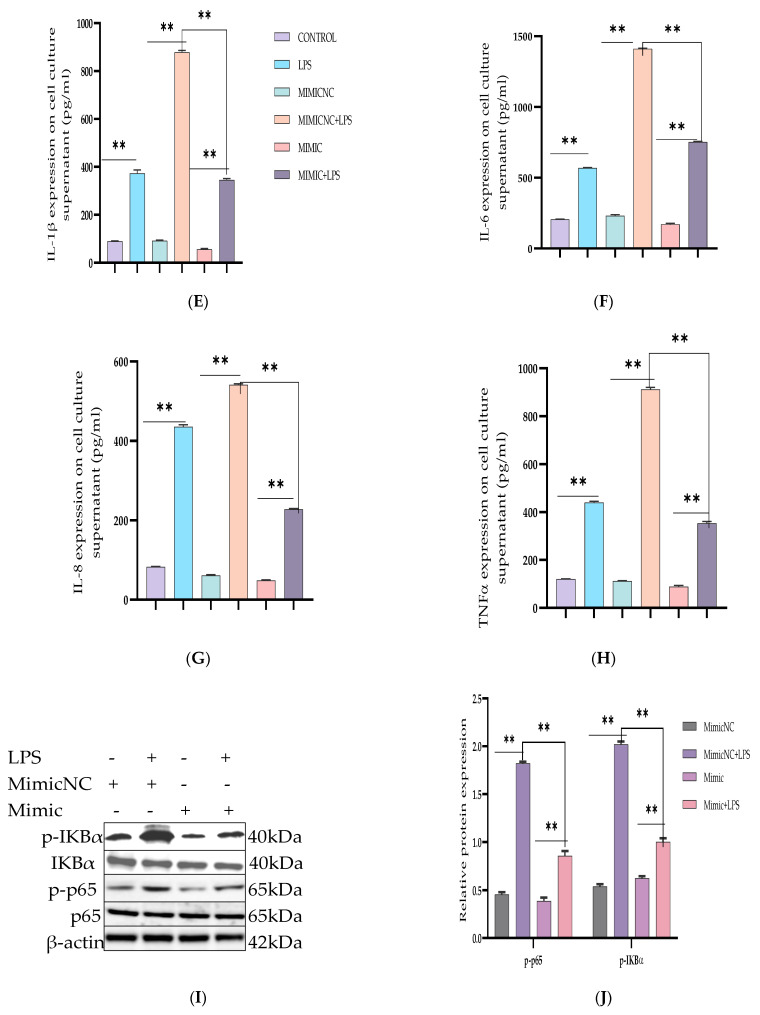

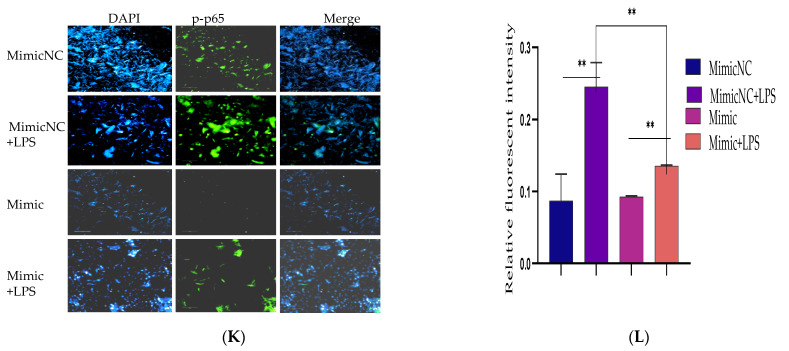
Overexpression of Bta-miR-24-3p attenuated LPS-induced inflammatory response. (**A**–**D**) Cells were transfected with 50 nM Bta-miR-24-3pmimics or 100 nM Bta-miR-24-3p NC for 7 h, and then stimulated with 3 µg/mL LPS for 24 h. The expression of cytokines IL-1β, IL-6, IL-8, and TNF-α was determined by RT-qPCR. β-actin was used as an endogenous control. (**E**,**H**) The cell supernatant from the transfection experiment was harvested to evaluate the cytokine concentration using the ELISA method. (**I**) BEECs was transfected with 50 nM Bta-miR-24-3p mimics or 100 nM Bta-miR-24-3p NC for 7 h and then stimulated with 3 µg/mL LPS for 36 h. The protein levels of NF-κB p65 and IκBα were measured by Western blotting. β-actin as an internal control. (**J**) The differential values of the indicated proteins were measured by IPP 6.0 software. (**K**) Immunofluorescence staining revealed the p65 subunit translocation from the cytoplasm into the nucleus (×200) using scale bar = 100 µm. cell nuclei were stained as blue spots, and green spots indicate p-p65 staining. (**L**) The relative fluorescence intensity of p-p65. The data were presented with triplicate experimental observations as mean ± SEM. * *p* < 0.05; ** *p* < 0.01. (Student *t*-test).

**Figure 3:**
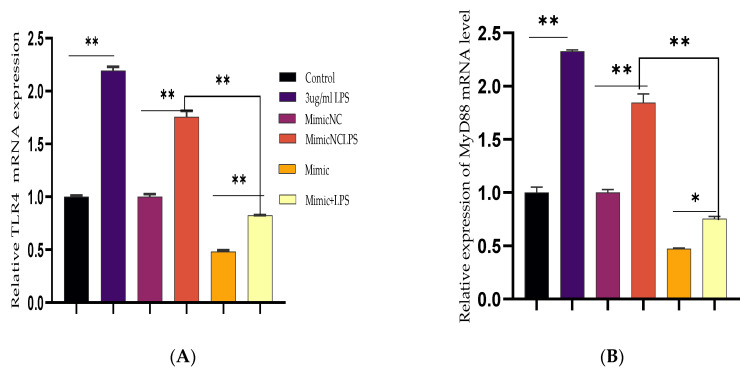

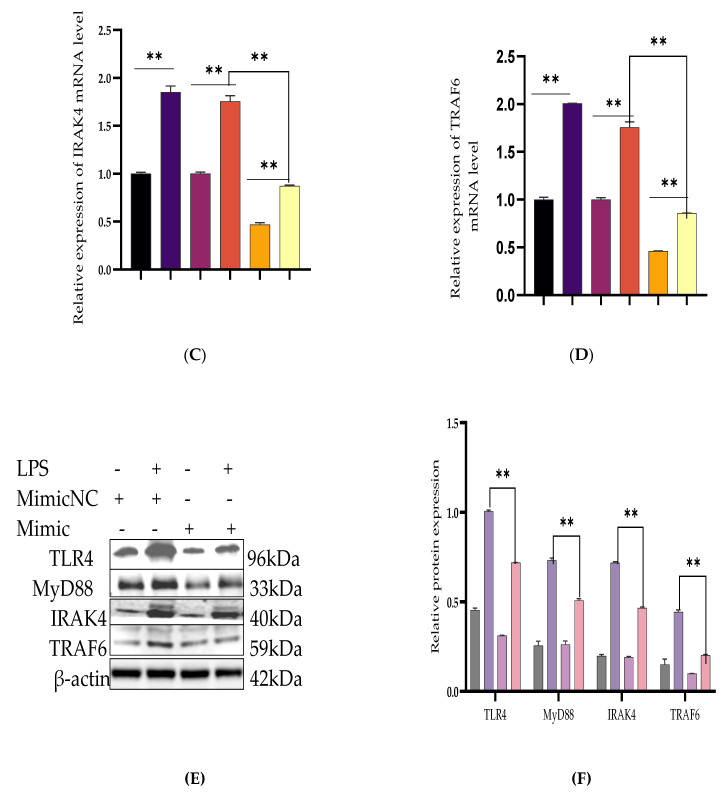
Downregulation of TLR4 and its effect modulator by Bta-miR-24-3p. BEECs were transfected with 50 nM of Bta-miR-24-3p mimics or 100 nM of Bta-miR-24-3p NC for 7 h and then stimulated with 3 µg/mL LPS for 24 h (**A**–**D**). The mRNA expression of TLR4, MyD88, IRAK4, and TRAF6 was determined by RT-qPCR. β-actin was used as an endogenous control. (**E**) Cells were treated as (**A**–**D**), and the protein levels of upstream molecules of the TLR4 pathway were measured by western blotting. (**F**) Gray values of the indicated proteins were measured by Image-Pro Plus (IPP) 6.0 software. The results are presented as the average of three experimental observations with mean ± SEM. * *p* < 0.05; ** *p* < 0.01. (Student *t*-test).

To further demonstrate the specific anti-inflammatory and inhibitory potentials of Bta-miR-24-3p in LPS-mediated inflammatory processes, Bta-miR-24-3p mimics were transiently transfected into BEECs. The expression level of pro-inflammatory cytokines was significantly increased (*p* < 0.01) by RT-qPCR assay upon transfection with Bta-miR-24-3p mimics for 24 h (Figure 2A–D). After transfection of cells with microRNA and LPS (3 μg/mL) as required, inflammatory cytokine concentration from the cell supernatant was determined by ELISA. As shown in Figure 2E–H, the concentration of pro-inflammatory cytokines was significantly suppressed on exposure to Bta-miR-24-3p with LPS-induction, which revealed a significant decrease (*p* < 0.01). Afterwards, to validate the role of Bta-miR-24-3p in the TLR4/NF-ĸB signaling pathway activation, protein samples from the transfection experiment were processed to determine the protein level of the TLR4/NF-ĸB signaling and their effector molecules using the Western blotting method and translocation of p-p65 using the Immunofluorescence assay. The phosphorylation levels ofIKBα and NF-κB p65 were deactivated significantly in Bta-miR-24-3p transfection, as shown in Figure 2I,J. Overexpression of Bta-miR-24-3p instead caused a significant decrease (*p* < 0.01) in their levels, indicating that Bta-miR-24-3p inhibited the phosphorylation of NF-κBp65.Moreover, immunofluorescence experiments were performed and demonstrated that Bta-miR-24-3p mimics had similar results post-transfection (Figure 2K,L). After transfection of Bta-miR-24-3p and required LPS-induction, the result revealed a significant decrease (*p* < 0.01) in the expression level of TLR4, MyD88, IRAK4, and TRAF6 (Figure 3A–D), which deduced the potential of Bta-miR-24-3p to suppression the activation of TLR4 through its receptor molecules. Nevertheless, mimic transfection significantly reduced TLR4, MyD88, IRAK4, and TRAF6 protein expression (Figure 3E,F). This summation revealed that Bta-miR-24-3p repressed TLR4 and its effectors’ expression and further inhibited LPS-induced activation of the TLR4/NF-ĸB signaling pathway, leading to inflammation attenuation in bovine endometrium.

### 3.3. Upregulation of Pro-Inflammatory Cytokines by Bta-miR-24-3p Inhibitor in BEECs

**Figure 4:**
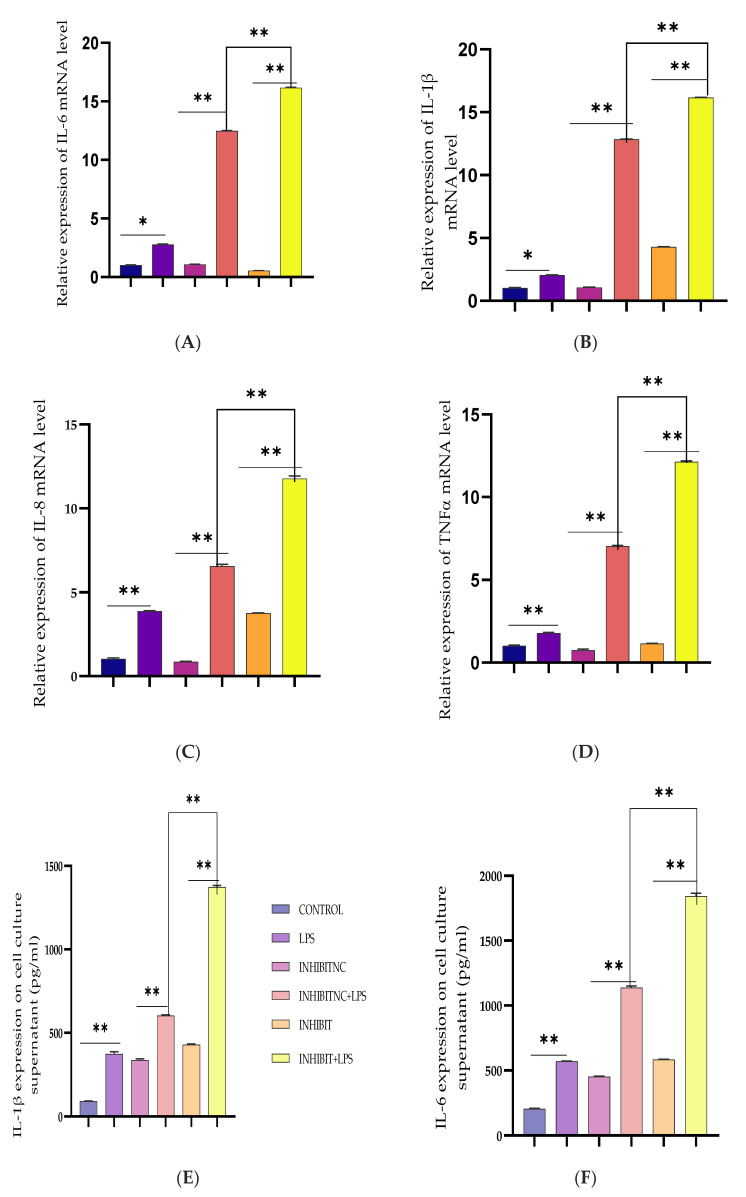

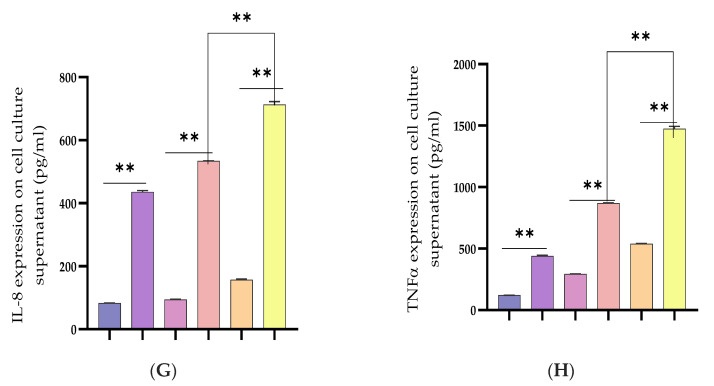
Inhibition of Bta-miR-24-3p elevated LPS-induced inflammatory response. (**A**–**D**) BEECs were transfected with 50 nM Bta-miR-24-3p inhibitors or 100 nM Bta-miR-24-3p inhibitors NC for 7 h and then stimulated with 3 µg/mL LPS for 24 h. The mRNA expression of cytokines (IL-1β, IL-6, IL-8, and TNF-α) was analysed using the RT-qPCR technique. β-actin was used as an endogenous control. (**E**–**H**) The ELISA technique assayed the pro-inflammatory cytokine concentration of IL-1β, IL-6, IL-8, and TNF-α in the cell supernatant. The results are presented as the average of three experimental observations with mean ± SEM. * *p* < 0.05; ** *p* < 0.01. (Student *t*-test).

BEECs were transiently transfected with the Bta-miR-24-3p inhibitors and negative control, and certain groups were then challenged with LPS. After transfection reaction, the expression level of pro-inflammatory cytokines was considerably elevated (*p* < 0.01) by RT-qPCR analysis (Figure 4A–D). ELISA was used to assess the concentration levels of inflammatory cytokines in the cell supernatant after transfection and treatment with LPS (3 μg/mL) as appropriate. LPS-mediated production of pro-inflammatory cytokines was considerably increased with exposure to Bta-miR-24-3p inhibitors, as shown in Figure 4E–H, with a significant rise in the concentration of the pro-inflammatory cytokines (*p* < 0.01). 

### 3.4. LGALS9 as a Direct Molecular Target of Bta-miR-24-3p

**Figure 5:**
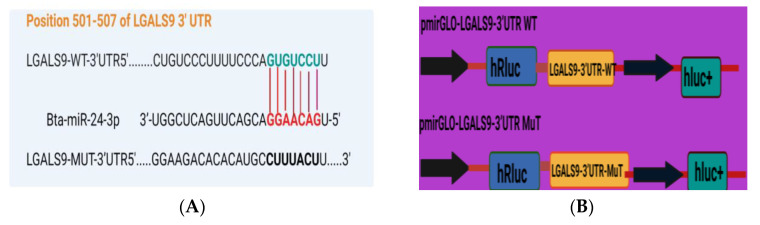

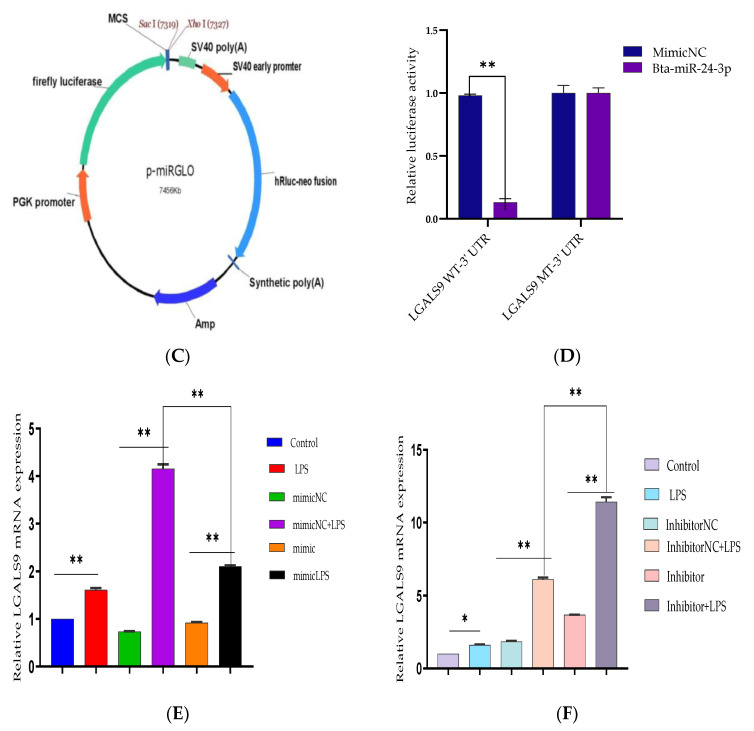
Evaluation of LGALS9 as a peculiar gene interacting with Bta-miR-24-3p. (**A**) The bioinformatics prediction of interaction between LGALS9-3′UTR and Bta-miR-24-3p was revealed by TargetScan and miRanda. (**B**) Recombinant cloning sketch of pmirGLO mutant-type LGALS9 3′-UTR or pmirGLO wild-type LGALS9 3′-UTRinto HEK293T cells. (**C**) Schematic construction of pmirGLO mutant-type LGALS9 3′-UTR and pmirGLO wild-type LGALS9 3′-UTR. (**D**) The dual-luciferase reporter assay was performed using HEK293T cells. Cells were co-transfected with the wild- or mutant-type LGALS9 3′-UTR luciferase reporter vectors, as well as Bta-miR-24-3p, mimics or mimics NC. The luciferase activity was represented by the ratio of Renilla activity/Firefly activity using the Luminometry method. (**E**) BEECs were transfected with 50 nM Bta-miR-24-3p mimics or 100 nM Bta-miR-24-3p mimics NC for 7 h and then stimulated with 3 µg/mL LPS for 24 h. The mRNA expression of LGALS9 was measured using the RT-qPCR technique. (**F**) BEECs were transfected with 50 nM Bta-miR-24-3p inhibitors or 100 nM Bta-miR-24-3p inhibitors NC for 7 h and then stimulated with 3 µg/mL LPS for 24 h. The mRNA expression of LGALS9 was measured using the RT-qPCR technique. β-actin was used as an endogenous control. The results are presented as the average of three experimental observations with mean ± SEM. * *p* < 0.05; ** *p* < 0.01. (Student *t*-test).

Based on TargetScan and miRanda findings, the LGALS9 3′-UTR contains a possible target site for Bta-miR-24-3p. The sequence of the target site was highly conserved in Bovine species (Figure 5A). The dual-luciferase recombinant, wild type, and mutant type plasmid sequences of LGALS9 3′-UTR are shown in Figure 5B,C. Recombinant plasmid with WT and MT LGALS9 3′-UTR were co-transfected with Bta-miR-24-3p mimic and NC, respectively, into HEK293T cells. Based on the dual-luciferase reporter assay, Bta-miR-24-3p significantly decreased (*p* < 0.05) the relative luciferase ratio in the pmirGLO-WT group compared to the mimic NC group. In contrast, there is no noticeable difference (*p* < 0.01) was found in the pmirGLO-MUT group (Figure 5D). The expression of LGALS9 in BEECs transfected with Bta-miR-24-3p mimic was significantly decreased (*p* < 0.01) as compared to the mimic NC group (Figure 5E), and the expression in the Bta-miR-24-3p inhibitor group was significantly increased (*p* < 0.01) compared with the inhibitor NC group (Figure 5F). Besides this, the expression of LGALS9 was decreased when Bta-miR-24-3p was overexpressed; these findings provide reasonable evidence that LGALS9 is a direct target gene of Bta-miR-24-3p and indicate that Bta-miR-24-3pmay have potential implications in inhibiting the expression of LGALS9 at the mRNA level.

### 3.5. Silencing LGALS9 Inhibits LPS-Mediated Inflammation in BEECs

**Figure 6:**
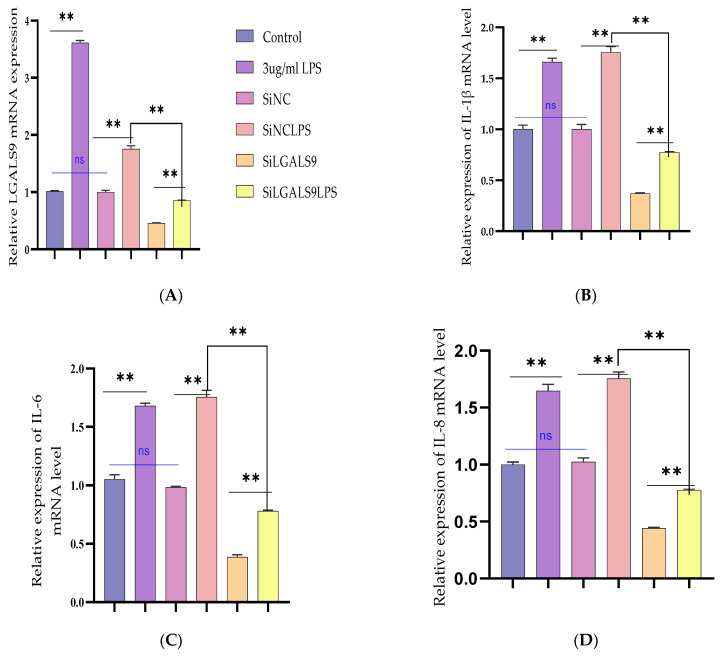

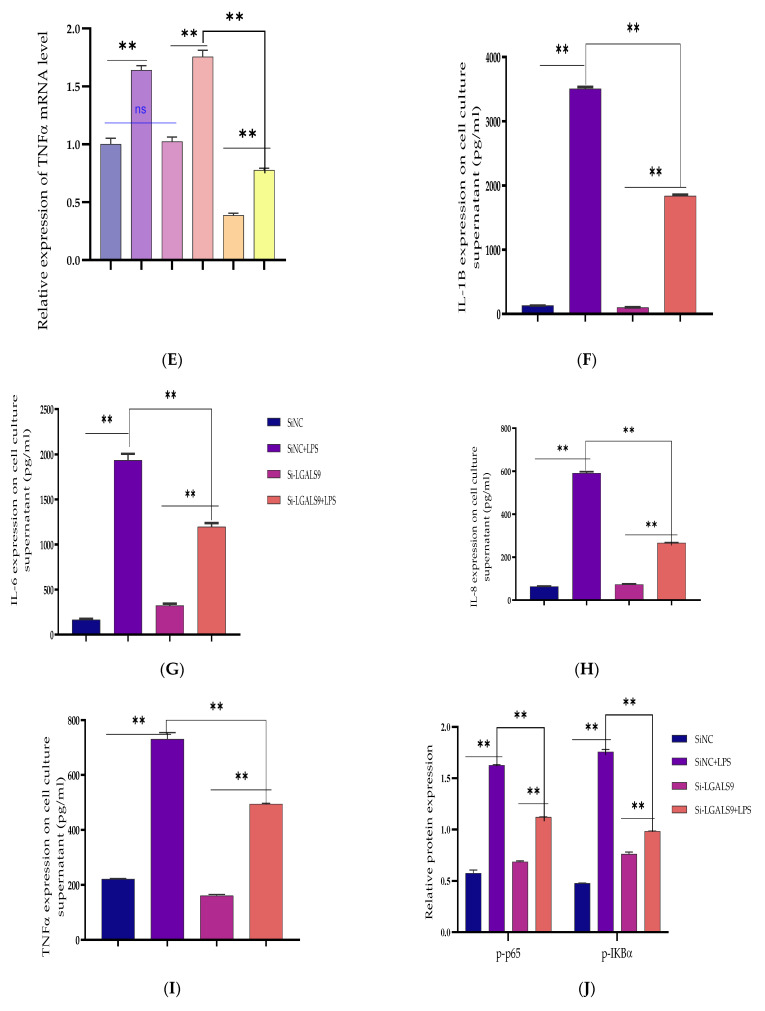

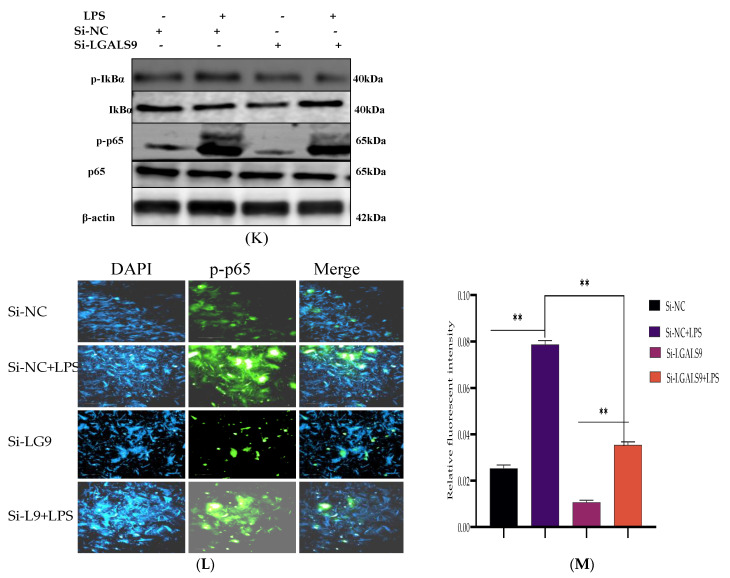
The cellular silencing of the LGALS9 gene in BEECs attenuates activation of pro-inflammatory cytokines and gene expression. The Si-LGALS9 and Si-NC were transfected for 7 h transfection and induction of BEECs with LPS (3 μg/mL) for another 24 h. (**A**) After the transfection of Si-LGALS9 and Si-NC, knockdown of LGALS9 (Si-LGALS9) results in LGALS9 downward expression using the RT-qPCR method. (**B**–**E**) The expression levels of pro-inflammatory mediators (IL-1β, IL-6, IL-8, and TNF-α) were analysed with the RT-qPCR technique. (**F**–**I**) The ELISA technique assayed the pro-inflammatory cytokine concentration of IL-1β, IL-6, IL-8, and TNF-α in the cell supernatant. (**J**–**K**) The protein phosphorylation levels of p65 and IκBα were measured by Western blot. β-actin was used as an internal control. (**L**–**M**) The fluorescence intensity of p-p65 protein expression using Immunofluorescence assay revealed reduced protein expression due to LGALS9 silencing. The scale bar is 100 μm. Data are represented by the mean ± SEM of triplicate independent experiments, ** *p* < 0.01. (Student *t*-test).

**Figure 7:**
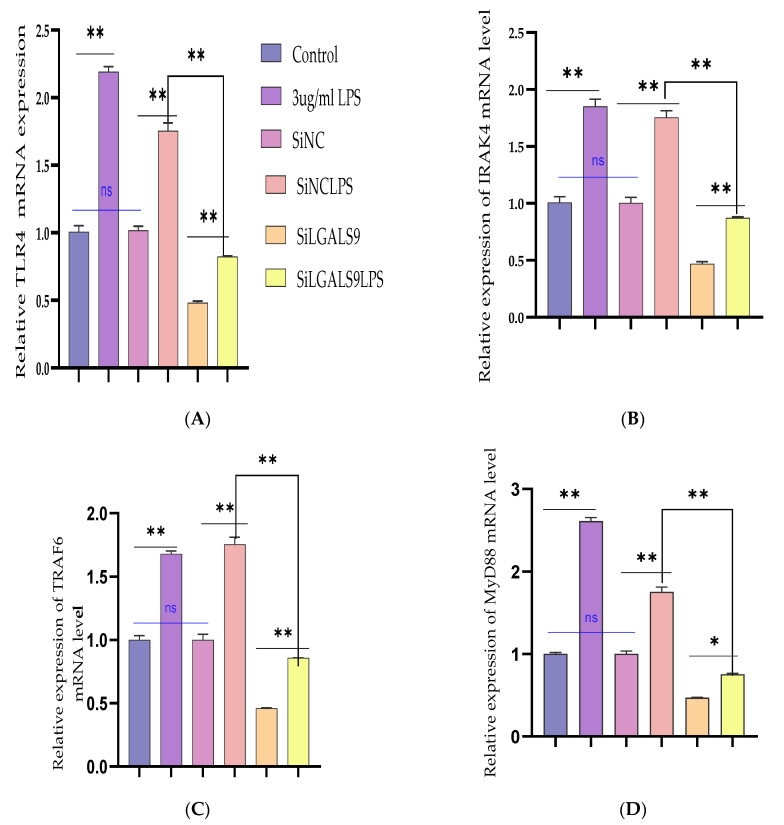
Silencing the expression of LGALS9 negatively modulates TLR4 and its effector molecules. The Si-LGALS9 and Si-NC have been transfected for 7 h of transfection and induction of BEECs with LPS (3 μg/mL) for another 24 h. (**A**–**D**) The mRNA expression levels of TLR4 and its molecular mediators (IRAK4, TRAF6, and MyD88) were analysed with the RT-qPCR technique. Data are represented by the mean ± SEM of triplicate independent experiments, * *p* < 0.05; ** *p* < 0.01. (Student *t*-test).

The need to expound the regulatory mechanism of Bta-miR-24-3p on LPS-mediated inflammatory responses needs to transfection of si-LGALS9 was knocked down by LGALS9 expression in BEECs compared with si-NC. The mRNA expression level of LGALS9 was downregulated by RT-qPCR experiments due to the downregulation of LGALS9, as shown in Figure 6A. The expression levels of the pro-inflammatory cytokines IL-1β, IL-6, IL-8, and TNF-α, were also inhibited following knockdown of LGALS9 (Figure 6B–E), which is consistent with the anti-inflammatory potential of Bta-miR-24-3p mimic. Furthermore, there was a reduction in the concentration of pro-inflammatory cytokines upon silencing LGALS9 in BEECs, as shown in Figure 6F–I. The knockdown of LGALS9 significantly inhibited the phosphorylation of NF-κBp65 and IKBα, similar to the above results in the Bta-miR-24-3p overexpression experiment (Figure 6J,K). In addition, Immunofluorescence staining also showed a similar phenomenon (Figure 6L,M). Also, the knockdown of LGALS9 in BEECs revealed a significant reduction (*p* < 0.01) in the mRNA expression level of TLR4 and its effectors (MyD88, IRAK4, and TRAF6), as shown in Figure 7A–D. Therefore, these findings clearly showed that the LPS-induced inflammatory response was associated with the negative regulation of the TLR4/NF-ĸB pathway by Bta-miR-24-3p targeting LGALS9.

### 3.6. Bta-miR-24-3p Regulates LPS-Induced Inflammatory Response by Targeting LGALS9

**Figure 8:**
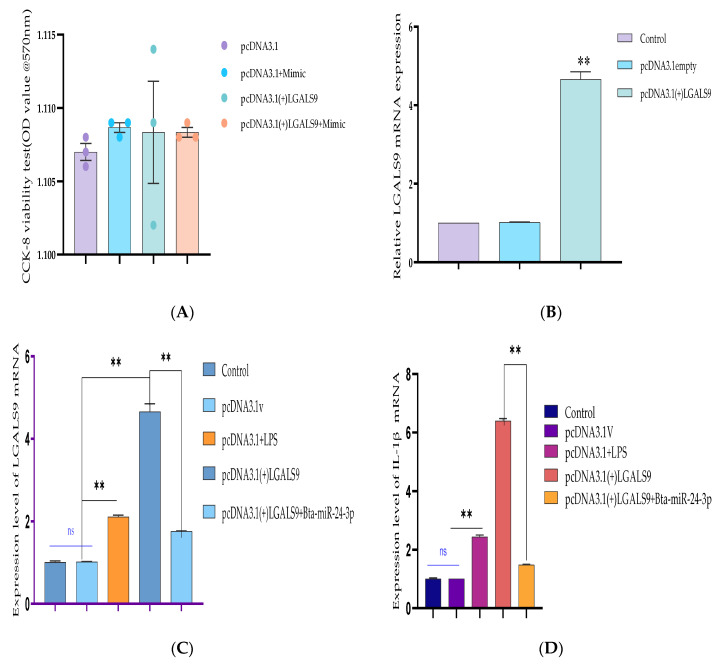

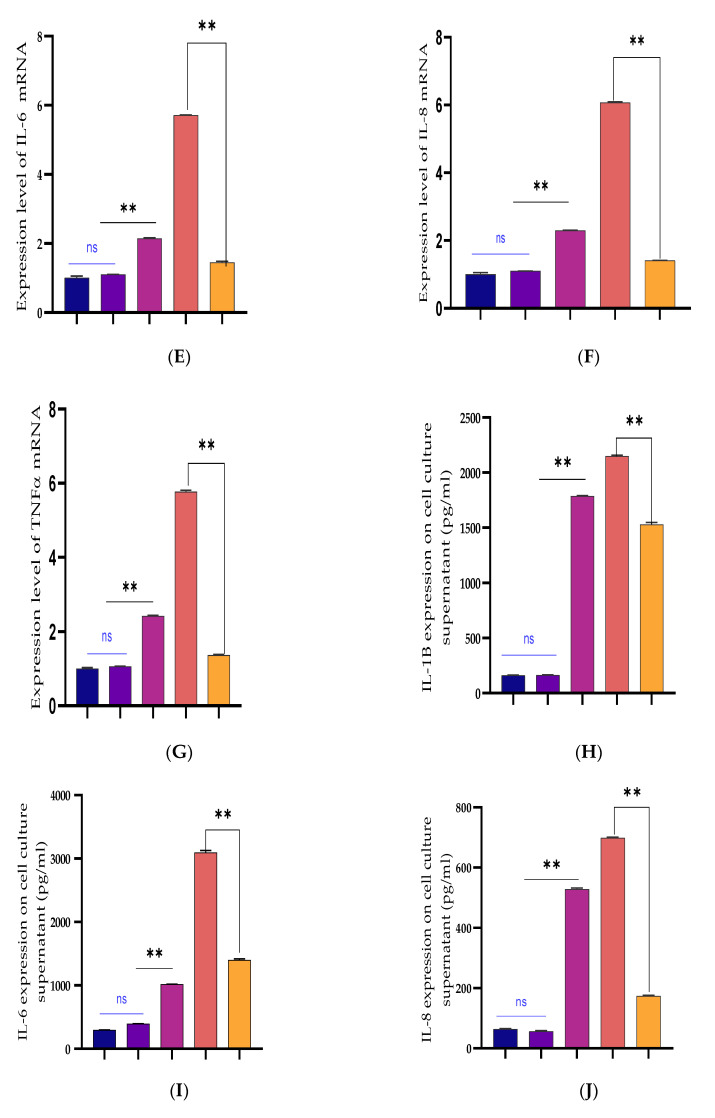

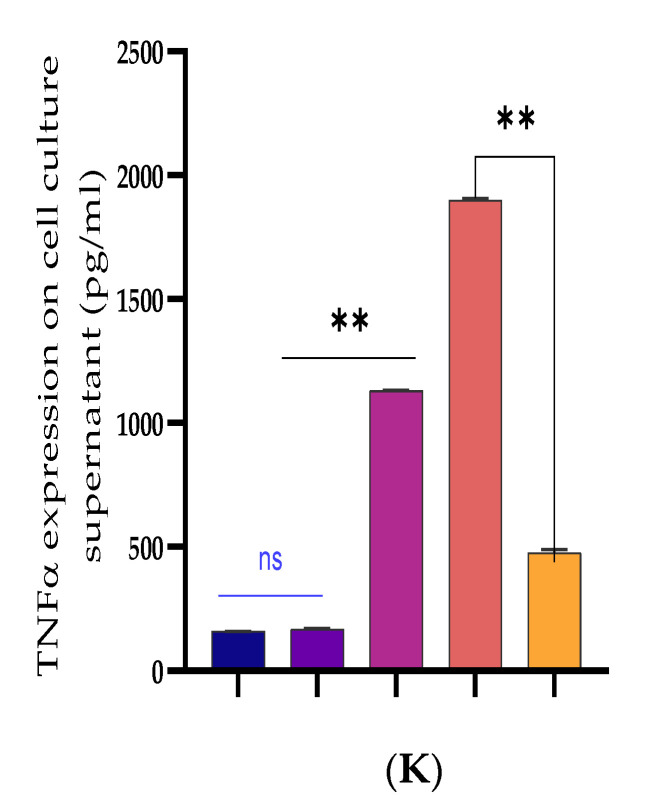
Recombinant DNA cloning vector construction and transfection into BEECs. (**A**) The evaluation of cell viability on pcDNA3.1empty vector, pcDNA3.1empty vector and Bta-miR-24-3p mimic, pcDNA3.1(+)TLR4, and pcDNA3.1(+)TLR4 and Bta-miR-24-3p mimic transfected into BEECs for 24 h was done using the CCK-8 assay kit. (**B**) The RT-qPCR was used to verify the transfection efficiency of pcDNA3.1empty vector and pcDNA3.1(+)TLR4 in BEECs (**C**). The expression level of LGALS9 was attenuated in co-transfection of pcDNA3.1(+)TLR4 and Bta-miR-24-3p mimics using the RT-qPCR technique. (**D**–**G**) Cells were transfected with pcDNA3.1empty vector, pcDNA3.1empty vector and Bta-miR-24-3p mimic, pcDNA3.1(+)TLR4, and pcDNA3.1(+)TLR4 and Bta-miR-24-3p for 24 h. The expression of cytokines IL-1β, IL-6, IL-8, and TNF-α was determined by RT-qPCR. β-actin was used as an endogenous control. (**H**–**K**) After the co-transfection experiment, the cell supernatants were collected to analyse the cytokine concentrations (IL-1β, IL-6, IL-8, and TNF-α) using the ELISA techniques. The results are presented as the average of three experimental observations with mean ± SEM. ** *p* < 0.01. (Student *t*-test).

**Figure 9:**
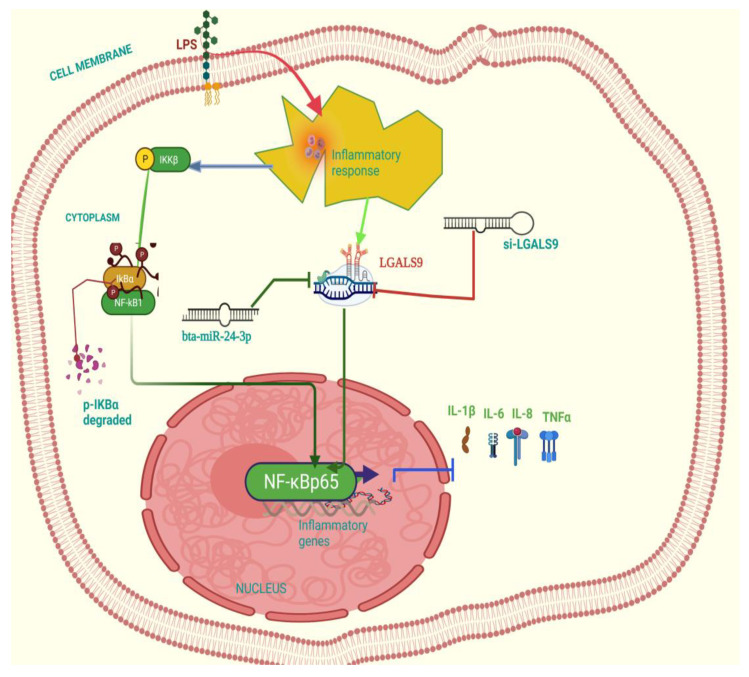
Schematic representation of the anti-inflammatory effect related signal transduction pathway in Bta-miR-24-3pon LPS-triggered bovine endometrial epithelial cells. This picture shows the induction of the cell with LPS to trigger an inflammatory response, which elevated the LGALS9 gene expression, and this gene expression was downregulated by Bta-miR-24-3p mimic. Likewise, knockdown of LGALS9 (si-LGALS9) leads to suppression of inflammatory response.

The recombinant DNA cloning vector of LGALS9 was constructed and schematically shown in Supplementary Figure S1 and transfected into BEECs. The CCK-8 cell viability assay result showed no significant difference among the pcDNA3.1 empty vector, pcDNA3.1 + Bta-miR-24-3pgroup, pcDNA3.1-LGALS9, and pcDNA3.1-LGALS9 and Bta-miR-24-3pgroups (*p* < 0.01) (Figure 8A). LGALS9 overexpression vector (pcdna3.1-LGALS9) was successfully generated to validate the regulating effect of LGALS9 on BEECs. LGALS9 mRNA expression levels increased significantly after transfecting BEECs with pcDNA3.1-LGALS9 (Figure 8B). However, the Bta-miR-24-3p mimic decreased LGALS9 expression, indicating that pcDNA3.1-LGALS9 might be employed in future investigations. The overexpression of Bta-miR24-3p decreased LGALS9 expression at mRNA levels; however, it could not be reversed after the co-transfection of Bta-miR24-3p mimics and LGALS9 (Figure 8C). Furthermore, in co-transfected BEECs, the restoration of LGALS9 could not counteract the anti-inflammatory effects of Bta-miR 24-3p on reducing the secretion of inflammatory cytokines (*p* < 0.01) (TNFα, IL1β, IL-8, and IL-6) (Figure 8D–G). The co-transfection of Bta-miR 24-3p mimic and pcDNA3.1-LGALS9 dramatically reduces the concentration of pro-inflammatory cytokines from the cell supernatants shown in Figure 8D–G (*p* < 0.01). These findings suggested that Bta-miR 24-3p could attenuate endometritis progression by inhibiting LGALS9 mRNA expression. The compilation of the research results has demonstrated the potential of Bta-miR-24-3p in regulating the LPS-induced bovine endometritis through attenuation of TLR4/NF-ĸB cellular signaling pathway by targeting LGALS9 (Figure 9).

## 4. Discussion

Endometritis is the significant implicated post-parturient uterine disease which slows down the follicular growth, distortion of ovulatory processes with the corresponding delay, and irregularity in the ovarian cycle leading to repeated breeding (reproductive failure), subfertility, and if not attended to, leads to infertility [2,5,44] and may lead to the extinction of dairy farms. Endometritis has been earlier termed a localized uterine lining infection resulting in sero-purulent with the trace of mucus discharge clinically and without any clearance sub clinically [8,9]. A significant factor is the massive influx of microorganisms, especially bacteria, into the uterine lumen during the term and early postpartum. The bacteria’s ease of invasion is aided by the endometrium anatomical position and physiological state, affecting postpartum endometrial functions [3,5,7]. Endometrial inflammation causes fertilisation problems, implantation failure, and early embryonic death [2,4,45] in dairy cows. Its failure causes ovarian cysts, luteal phase elongation, and postpartum estrus return failure [46,47,48]. The non-specific and local-specific anti-infective immunity of the uterus’s cellular and molecular mechanisms was evaluated, and endometrial cells have been the most referenced in the pathogenesis of endometritis [49]; thus, the need to explore the cellular component of the endometritis has been on top gear in the last few decades.

LPS is an endotoxin usually found on the surface of Gram-negative bacteria, engages through pro-inflammatory receptors and pathways, compromises endometrial cell function, and plays a crucial role in endometritis pathogenesis.LPS has been shown to cause implantation failure and pregnancy losses [13,14,15,17]. When LPS binds to toll-like receptors, it activates pathways that produce pro-inflammatory cytokines [50,51,52]. LPS significantly impacts many transcriptome markers associated with implantation and embryo maternal interactions, including the Galectin9 (LGALS9) gene [13]. Our report corroborates this due to the elevation of LGALS9 genes upon LPS-stimulation of Bovine Endometrial Epithelial Cells (BEECs). Likewise, LGALS9 was earlier reported as a significant gene that was highly upregulated in LPS-induced endometritis [13,14,15,16,17]. All uterine cell types, including endometrial epithelial and endothelial cells, express LGALS9 [18,20]. Galectin families are required to create an immune-privileged local milieu for implantation and early fetal development, as established in humans and rodents, owing to their immunosuppressive activities and a vital role in maternal-fetal tolerance [19,20,21]. The need to regulate the expression of LGALS9 during the window of implantation and in normal early decidual was essential in embryonic development; therefore, there is a need to curb premature embryonic death as the result of endometritis. It requires adequate evaluation to resolve this lingering endometritis [19,20,22,24]. Norling et al., 2009 [25] reported that overexpression of LGALS9 was linked to an increase in the transcription of several genes. LGALS9 may positively control the synthesis of pro-inflammatory cytokines in the shortage of immune cells, according to genes encoding for these molecules [13,15,26].

Many microRNAs have been implicated in endometritis pathogenesis in a quest to resolve the dairy industry menace [53], including miR-24. In this research, the stable transcript of miR-24 was used to evaluate its role in endometritis. Hu et al., 2019 [54] reported that Bta-miR-24-3p inhibits proliferation and promotes myogenic differentiation of progenitor cells in Fetal Bovine Skeletal Muscle. MiR-24-3p was also revealed to protect against myocardial ischemia and reperfusion injury by acting as a cardioprotective factor [55]. MiR-24 inhibits the proliferation and migration of vascular endothelial cells by deactivating the NF-ĸB signaling pathway, regulating inflammation in endothelial cells, and preventing the NF-ĸB signaling pathway in atherosclerosis [56]. In LPS-challenged neonatal rats, miR-24 overexpression reduced lung inflammation [57] and disrupted the inflammatory pathway [58]. In addition, some microRNA such as miR-19a [41], miR-148a [28], miR-98 [59], and others have been reported to attenuate the inflammatory effect of LPS on endometrial cells through various signaling pathways and receptors, especially TLR4/NF-ĸB signal pathways [28,41,59]. Thus, discovering a novel molecule inhibitor of LGALS9 and probable overexpression of Bta-miR-24-3p that targets specific receptors and pathways could offer a new path for developing selective inflammatory mediator blockers with potential therapeutic benefits wide range of inflammatory diseases. This study revealed that upon stimulation of BEECs with LPS, there was an elevation in the expression level of LGALS9 with a corresponding decrease in the level of Bta-miR-24-3p, indicating the alternate role of both in the molecular pathogenesis of endometritis. The transient upregulation of Bta-miR-24-3p into BEECs leads to downward regulation of LGALS9 and pro-inflammatory cytokines expression (IL-1β, IL-6, IL-8, and TNF-α).

Furthermore, the expression of TLR4 in the endometrium of postpartum cattle that develop endometritis and infertility has been affirmed to be higher by previous reports [42]; hence the potential suppressor of TLR4 and its effectors molecules could serve as a pointer to drug discovery against endometritis and other inflammatory condition. In our study, overexpression of Bta-miR-24-3p remarkably decreased the expression level of the TLR4, MyD88, IRAK4, and TRAF6 genes and protein levels in LPS-stimulated BEECs, which means the Bta-miR-24-3p, could regulate the down-stream transduction of TLR4 in inflammatory diseases. Ultimately, LGALS9 was affirmed as a molecular target gene of Bta-miR-24-3p through bioinformatics prediction and experimental confirmation with a notable decrease in dual-luciferase activity upon Bta-miR-24-3p mimics transfection. NF-ĸB activation stimulates the transcription of inflammatory mediator genes involved in the incidence and development of endometritis [24,60]. To further understand the molecular processes by which Bta-miR24-3p inhibits the generation of pro-inflammatory cytokines, we investigated the effect of Bta-miR24-3p on NF-ĸB pathway activation. Based on our assumption, Bta-miR24-3p was found to attenuate the phosphorylation of NF-ĸBp65 and IKBα; therefore, overexpression of Bta-miR24-3p could suppress LPS-induced endometritis, probably via inhibiting the NF-ĸB pathway’s activation. Researchers have earlier observed that activation ofTLR4through recruitment of its adaptor molecules, such as MyD88, IRAK4, and TRAF6, often leads to activation of the NF-ĸBsignaling pathway. It recognized that Bta-miR-24-3p regulates LPS-triggered endometritis by suppressing the TLR4/NF-ĸB signaling pathway by targeting the LGALS9 gene.

LGALS9 is an endometrial gene found to incriminate in the pathogenesis of endometritis, leading to some other reproductive problems [18,19,20]. Overexpression of LGALS9 was reported in LPS-stimulated endometritis, depicting its role in the pathophysiology of bovine endometritis [13,15]. The need to knock down the expression of LGALS9 in BEECs could portray a point biomarker as molecular therapeutic regulation of endometritis. Experimental transient downregulation of LGALS9 in BEECs with LPS-stimulation revealed the suppression of pro-inflammatory cytokines, and LGALS9 with the deactivation of TLR4/NF-ĸB signaling pathway has been observed in overexpression of Bta-miR-24-3p. Additionally, recombinant cloning of LGALS9 into BEECs experiments confirmed that downward regulation of LGALS9 could enhance the therapeutic effect of Bta-miR-24-3p on LPS-induced endometritis, indicating that Bta-miR-24-3p could regulate endometritis progression by downregulating LGALS9.

## 5. Conclusions

In conclusion, for the first time, this study revealed the cellular and molecular regulatory function of Bta-miR-24-3p against endometritis by targeting the LGALS9 gene. The modulation of signaling pathways and receptors by Bta-miR-24-3p could serve as a therapeutic basement against inflicting inflammatory reactions.

## Data Availability

This article has included all the data generated and analyzed in this study. Supplementary information is attached in a separate file.

## Supplementary Materials

The following are available online at https://www.mdpi.com/article/10.3390/cells10123299/s1, Figures S1–S3: show the picture of bovine endometrial epithelial cells, Figure S4: presents the constructed plasmid DNA, Table S1: show the sequences of the RNA Oligonucleotide, Table S2: show primer sequences for the inflammatory cytokines and LGALS9 for RT-qPCR.

## References

[1] Sheldon I.M., Cronin J., Borges A. (2011). The Postpartum Period and Modern Dairy Cow Fertility. Part 1: Uterine Function. Livestock.

[2] Gilbert R.O. (2012). The Effects of Endometritis on the Establishment of Pregnancy in Cattle. Reprod. Fertil. Dev..

[3] Leblanc S., Duffield T., Leslie K., Bateman K., Keefe G., Walton J., Johnson W. (2002). Defining and Diagnosing Postpartum Clinical Endometritis and its Impact on Reproductive Performance in Dairy Cows. J. Dairy Sci..

[4] Gilbert R.O., Shin S.T., Guard C.L., Erb H.N., Frajblat M. (2005). Prevalence of Endometritis and its Effects on Reproductive Performance of Dairy Cows. Theriogenology.

[5] Borsberry S., Dobson H. (1989). Periparturient Diseases and their Effect on Reproductive Performance in Five Dairy Herds. Veter. Rec..

[6] Minten M.A., Bilby T.R., Bruno R.G.S., Allen C.C., Madsen C.A., Wang Z., Sawyer J., Tibary A., Neibergs H.L., Geary T.W. (2013). Effects of Fertility on Gene Expression and Function of the Bovine Endometrium. PLoS ONE.

[7] Bonneville-Hébert A., Bouchard E., Du Tremblay D., Lefebvre R. (2011). Effect of Reproductive Disorders and Parity on Repeat Breeder Status and Culling of Dairy Cows in Quebec. Can. J. Veter. Res. Rev. Can. Rech. Veter..

[8] Yusuf M., Nakao T., Ranasinghe R.B.K., Gautam G., Long S.T., Yoshida C., Koike K., Hayashi A. (2010). Reproductive Performance of Repeat Breeders in Dairy Herds. Theriogenology.

[9] Salasel B., Mokhtari A., Taktaz T. (2010). Prevalence, Risk Factors for and Impact of Subclinical Endometritis in Repeat Breeder Dairy Cows. Theriogenology.

[10] Janowski T., Barański W., Łukasik K., Skarzynski D.J., Rudowska M., Zduńczyk S. (2013). Prevalence of Subclinical Endometritis in Repeat Breeding Cows and mRNA Expression of Tumor Necrosis Factor α and Inducible Nitric Oxide Synthase in the Endometrium of Repeat Breeding Cows with and Without Subclinical Endometritis. Pol. J. Veter. Sci..

[11] Mesquita F.S., Ramos R.S., Pugliesi G., Andrade S., Van Hoeck V., Langbeen A., Oliveira M.L., Gonella-Diaza A.M., Gasparin G., Fukumasu H. (2015). The Receptive Endometrial Transcriptomic Signature Indicates an Earlier Shift from Proliferation to Metabolism at Early Diestrus in the Cow1. Biol. Reprod..

[12] Jaiswal M., LaRusso N.F., Burgart L.J., Gores G.J. (2000). Inflammatory Cytokines Induce DNA Damage and Inhibit DNA Repair in Cholangiocarcinoma Cells by a Nitric Oxide-Dependent Mechanism. Cancer Res..

[13] Oguejiofor C., Cheng Z., Abudureyimu A., Fouladi-Nashta A.A., Wathes D.C. (2015). Global Transcriptomic Profiling of Bovine Endometrial Immune Response In Vitro. I. Effect of Lipopolysaccharide on Innate Immunity1. Biol. Reprod..

[14] Granot I., Gnainsky Y., Dekel N., Jiménez-Trejo F., Tapia-Rodríguez M., Cerbón M., Kuhn D.M., Manjarrez-Gutiérrez G., Mendoza-Rodríguez C.A., Picazo O. (2012). Endometrial Inflammation and Effect on Implantation Improvement and Pregnancy Outcome. Reproduction.

[15] Yongzhi G., Tom V.S., Jhamat N., Adnan Niaz C.M., Gilles Charpigny V.J.F., Erik B., Patrice H. (2019). Differential Gene Expression in Bovine Endometrial Epithelial Cells after Challenge with LPS; Specific Implications for Genes Involved in Embryo Maternal Interactions. PLoS ONE.

[16] Fortier M.A., Guilbault L.A., Grasso F. (1988). Specific Properties of Epithelial and Stromal Cells from the Endometrium of Cows. Reproduction.

[17] Gómez-Chávez F., Castro-Leyva V., Espejel-Núñez A., Zamora-Mendoza R.G., Rosas-Vargas H., Cancino-Díaz J.C., Cancino-Díaz M.E., Estrada-Gutierrez G., Rodríguez-Martínez S. (2015). Galectin-1 Reduced the Effect of LPS on the IL-6 Production in Decidual Cells by Inhibiting LPS on the Stimulation of IκBζ. J. Reprod. Immunol..

[18] Heusschen R., Griffioen A.W., Thijssen V. (2013). Galectin-9 in Tumor Biology: A Jack of Multiple Trades. Biochim. Biophys. Acta (BBA) Bioenerg..

[19] Shimizu Y., Kabir-Salmani M., Azadbakht M., Sugihara K., Sakai K., Iwashita M. (2008). Expression and Localization of Galectin-9 in the Human Uterodome. Endocr. J..

[20] Heusschen R., Freitag N., Tirado-Gonzalez I., Barrientos G., Moschansky P., Muñoz-Fernández R., Leno-Duran E., Klapp B.F., Thijssen V.L., Blois S.M. (2013). Profiling Lgals9 Splice Variant Expression at the Fetal-Maternal Interface: Implications in Normal and Pathological Human Pregnancy1. Biol. Reprod..

[21] Popovici R.M., Krause M.S., Germeyer A., Strowitzki T., von Wolff M. (2005). Galectin-9: A New Endometrial Epithelial Marker for the Mid- and Late-Secretory and Decidual Phases in Humans. J. Clin. Endocrinol. Metab..

[22] Thijssen V.L., Hulsmans S., Griffioen A.W. (2008). The Galectin Profile of the Endothelium: Altered Expression and Localization Inactivated and Tumor Endothelial Cells. Am. J. Pathol..

[23] Froehlich R., Hambruch N., Haeger J.-D., Dilly M., Kaltner H., Gabius H.-J., Pfarrer C. (2012). Galectin Fingerprinting Detects Differences in Expression Profiles between Bovine Endometrium and Placentomes as Well as Early and Late Gestational Stages. Placenta.

[24] Mitko K.G. (2008). Dynamic Transcriptome Profiling of Bovine Endometrium during the Oestrous Cycle. Master’s Thesis.

[25] Norling L.V., Perretti M., Cooper D. (2009). Endogenous Galectins and the Control of the Host Inflammatory Response. J. Endocrinol..

[26] Piersanti R.L., Block J., Ma Z., Jeong K.C., Santos J.E.P., Yu F., Sheldon I.M., Bromfield J.J. (2020). Uterine Infusion of Bacteria Alters the Transcriptome of Bovine Oocytes. FASEB BioAdv..

[27] Brinchmann M.F., Patel D.M., Iversen M.H. (2008). Review Article the Role of Galectins as Modulators of Metabolism and Inflammation. Mediat. Inflamm..

[28] Jiang K., Yang J., Yang C., Zhang T., Shaukat A., Yang X., Dai A., Wu H., Deng G. (2020). miR-148a Suppresses Inflammation in Lipopolysaccharide-Induced Endometritis. J. Cell. Mol. Med..

[29] Sheldon I.M., Cronin J., Goetze L., Donofrio G., Schuberth H.-J. (2009). Defining Postpartum Uterine Disease and the Mechanisms of Infection and Immunity in the Female Reproductive Tract in Cattle1. Biol. Reprod..

[30] Gonzalez-Ramos R., Defrere S., Devoto L. (2012). Nuclear Factor-kappaB: A Main Regulator of Inflammation and Cell Survival in Endometriosis Pathophysiology. Fertil. Steril..

[31] Kagan J.C., Medzhitov R. (2006). Phosphoinositide-Mediated Adaptor Recruitment controls Toll-Like Receptor Signaling. Cell.

[32] Torchinsky A., Markert U.R., Toder V. (2005). TNF-&agr;-Mediated Stress-Induced Early Pregnancy Loss: A Possible Role of Leukemia Inhibitory Factor. Anaphylaxis.

[33] Piras C., Guo Y., Soggiu A., Chanrot M., Greco V., Urbani A., Charpigny G., Bonizzi L., Roncada P., Humblot P. (2017). Changes in Protein Expression Profiles in Bovine Endometrial Epithelial Cells Exposed to E. Coli LPS Challenge. Mol. BioSyst..

[34] Ibeagha-Awemu E., Lee J.-W., Ibeagha A.E., Bannerman D.D., Paape M.J., Zhao X. (2008). Bacterial Lipopolysaccharide induces Increased Expression of Toll-Like Receptor (TLR) 4 and Downstream TLR Signaling Molecules in Bovine Mammary Epithelial Cells. Veter. Res..

[35] Cronin J.G., Turner M.L., Goetze L., Bryant C.E., Sheldon I.M. (2012). Toll-Like Receptor 4 and MYD88-Dependent Signaling Mechanisms of the Innate Immune System are Essential for the Response to Lipopolysaccharide by Epithelial and Stromal Cells of the Bovine Endometrium. Biol. Reprod..

[36] Sheldon I.M., Roberts M.H. (2010). Toll-Like Receptor 4 Mediates the Response of Epithelial and Stromal Cells to Lipopolysaccharide in the Endometrium. PLoS ONE.

[37] Meijer H.A., Kong Y.W., Lu W.T., Wilczynska A., Spriggs R.V., Robinson S.W., Godfrey J.D., Willis A.E., Bushell M. (2013). Translational Repression and Eif4a2 Activity are Critical for Microrna-Mediated Gene Regulation. Science.

[38] Bartel D.P. (2004). MicroRNAs: Genomics, Biogenesis, Mechanism, and Function. Cell.

[39] Chekulaeva M., Filipowicz W. (2009). Mechanisms of miRNA-Mediated Post-Transcriptional Regulation in Animal Cells. Curr. Opin. Cell Biol..

[40] Ambros V. (2004). The Functions of Animal MicroRNAs. Nature.

[41] Yin N., Yang Y., Wang X., Yang C., Ma X., Shaukat A., Zhao G., Deng G. (2019). MiR-19a Mediates the Negative Regulation of the NF-Κb Pathway in Lipopolysaccharide-Induced Endometritis by Targeting TBK1. Inflamm. Res..

[42] Herath S., Lilly S.T., Santos N.R., Gilbert O.R., Goetze L., Bryant C.E., White O.J., Cronin J., Sheldon I.M. (2009). Expression of Genes Associated with Immunity in the Endometrium of Cattle with Disparate Postpartum Uterine Disease and Fertility. Reprod. Biol. Endocrinol..

[43] Kim V.N., Nam J.W. (2006). Genomics of microRNA. Trends Genet..

[44] Sponchiado M., Gomes N.S., Fontes P., Martins T., del Collado M., Pastore A.D.A., Pugliesi G., Nogueira M.F.G., Binelli M. (2017). Pre-Hatching Embryo-Dependent and -Independent Programming of Endometrial Function in Cattle. PLoS ONE.

[45] Lee J.-I., Kim I.-H. (2007). Pregnancy Loss in Dairy Cows: The Contributing Factors, the Effects on Reproductive Performance and the Economic Impact. J. Vet. Sci..

[46] Herath S., Lilly S.T., Fischer D.P., Williams E.R., Dobson H., Bryant C.E., Sheldon I.M. (2009). Bacterial Lipopolysaccharide Induces an Endocrine Switch from Prostaglandin F2alpha to Prostaglandin E2 in Bovine Endometrium. Endocrinology.

[47] Mateus L., Lopes-Da-Costa L., Carvalho H., Serra P., Silva J.R. (2002). Blood and Intrauterine Leukocyte Profile and Function in Dairy Cows that Spontaneously Recovered from Postpartum Endometritis. Reprod. Domest. Anim..

[48] Sheldon I., Dobson H. (2004). Postpartum Uterine Health in Cattle. Anim. Reprod. Sci..

[49] Turner M., Healey G., Sheldon I.M. (2012). Immunity and Inflammation in the Uterus. Reprod. Domest. Anim..

[50] Akira S., Uematsu S., Takeuchi O. (2006). Pathogen Recognition and Innate Immunity. Cell.

[51] Akira S., Takeda K. (2004). Toll-Like Receptor Signaling. Nat. Rev. Immunol..

[52] Beutler B. (2004). Inferences, Questions, and Possibilities in Toll-Like Receptor Signaling. Nature.

[53] Hailemariam D., Ibrahim S., Hoelker M., Drillich M., Heuwieser W., Looft C., Cinar M.U., Tholen E., Schellander K., Tesfaye D. (2014). MicroRNA-Regulated Molecular Mechanism Underlying Bovine Subclinical Endometritis. Reprod. Fertil. Dev..

[54] Hu X., Xing Y., Ren L., Wang Y., Li Q., Fu X., Yang Q., Xu L., Willems L., Li J. (2019). Bta-miR-24-3p Controls the Myogenic Differentiation and Proliferation of Fetal, Bovine, Skeletal Muscle-Derived Progenitor Cells by Targeting ACVR1B. Animals.

[55] Tan H., Qi J., Fan B.-Y., Zhang J., Su F.-F., Wang H.-T. (2018). MicroRNA-24-3p Attenuates Myocardial Ischemia/Reperfusion Injury by Suppressing RIPK1 Expression in Mice. Cell. Physiol. Biochem..

[56] Zheng Y., Li Y., Liu G., Qi X., Cao X. (2018). MicroRNA-24 Inhibits the Proliferation and Migration of Endothelial Cells in Patients with Atherosclerosis by Targeting Importin-A3 and Regulating Inflammatory Responses. Exp. Ther. Med..

[57] Lin Y., Yang Y. (2019). MiR-24 Inhibits Inflammatory Responses in LPS-Induced Acute Lung Injury of Neonatal Rats through Targeting NLRP3. Pathol. Res. Pract..

[58] Sun Q., Zhang Y., Yang G., Chen X., Zhang Y., Cao G., Wang J., Sun Y., Zhang P., Fan M. (2008). Transforming Growth Factor-Beta-Regulated Mir-24 Promotes Skeletal Muscle Differentiation. Nucleic Acids Res..

[59] Nakamura K., Kusama K., Ideta A., Kimura K., Hori M., Imakawa K. (2019). Effects of miR-98 in Intrauterine Extracellular Vesicles on Maternal Immune Regulation during the Peri-Implantation Period in Cattle. Sci. Rep..

[60] Jiang K., Guo S., Yang J., Liu J., Shaukat A., Zhao G., Wu H., Deng G. (2019). Matrine Alleviates Staphylococcus Aureus Lipoteichoic Acid-Induced Endometritis via Suppression of TLR2-Mediated NF-κB Activation. Int. Immunopharmacol..

