# Supplementary material for: MicroRNA Bta-miR-24-3p Suppressed Galectin-9 Expression through TLR4/NF-ĸB Signaling Pathway in LPS-Stimulated Bovine Endometrial Epithelial Cells
Source: Cells. 2021 Nov 25;10(12):3299. doi: 10.3390/cells10123299 (PMC8699331; doi:10.3390/cells10123299)
Supplement: Supplementary file 1 [file cells-10-03299-s001.zip › cells-1447727-supplementary.pdf]

Supplementary Figures S1–S3:

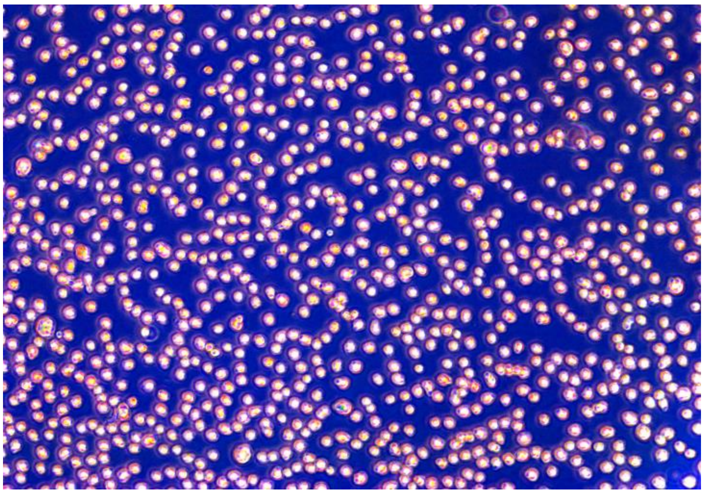

Dissociated endometrial epithelial cells

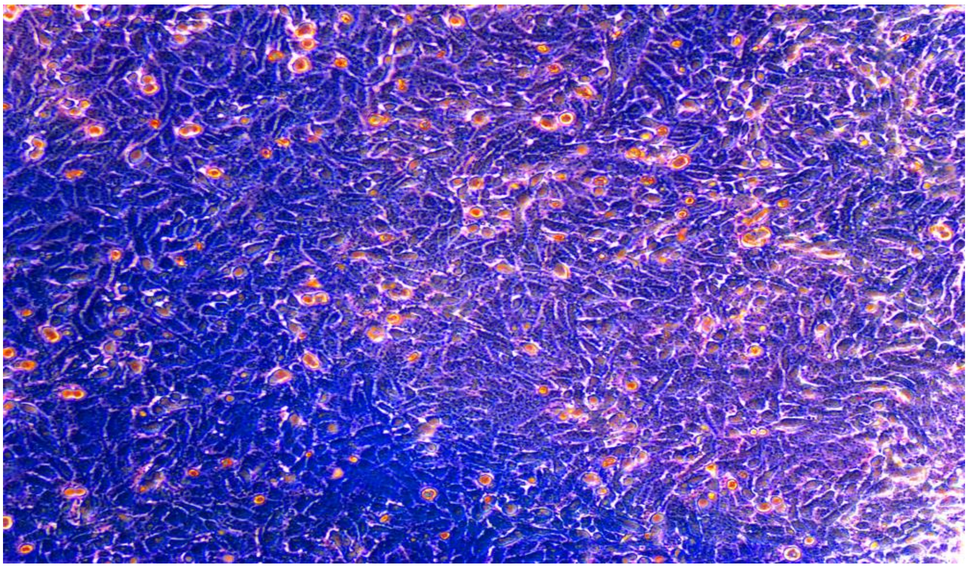

Adherent culture endometrial epithelial cells (densely populated cells)

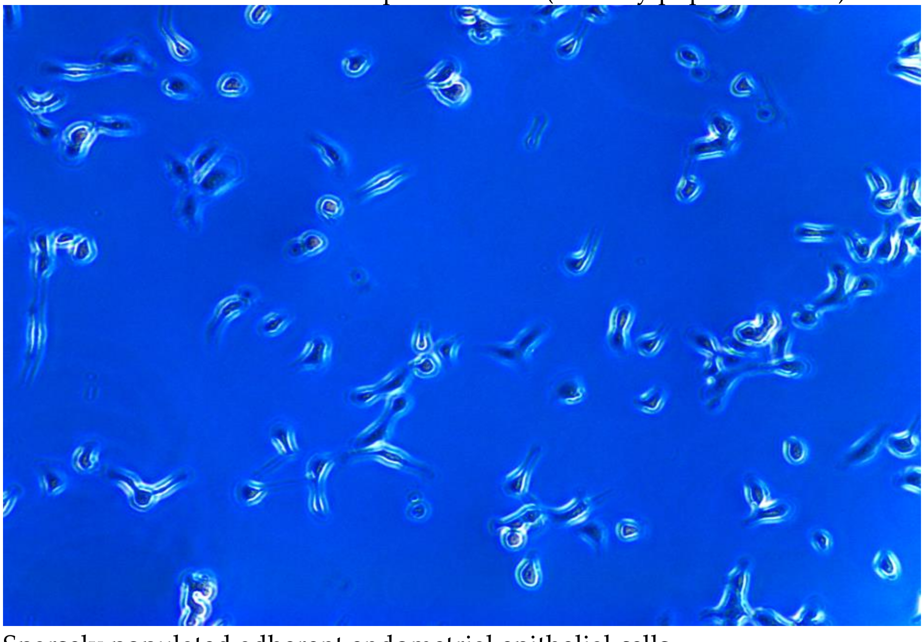

Sparsely populated adherent endometrial epithelial cells

Supplementary Figure S4. The recombinant DNA cloning vector of LGALS9.

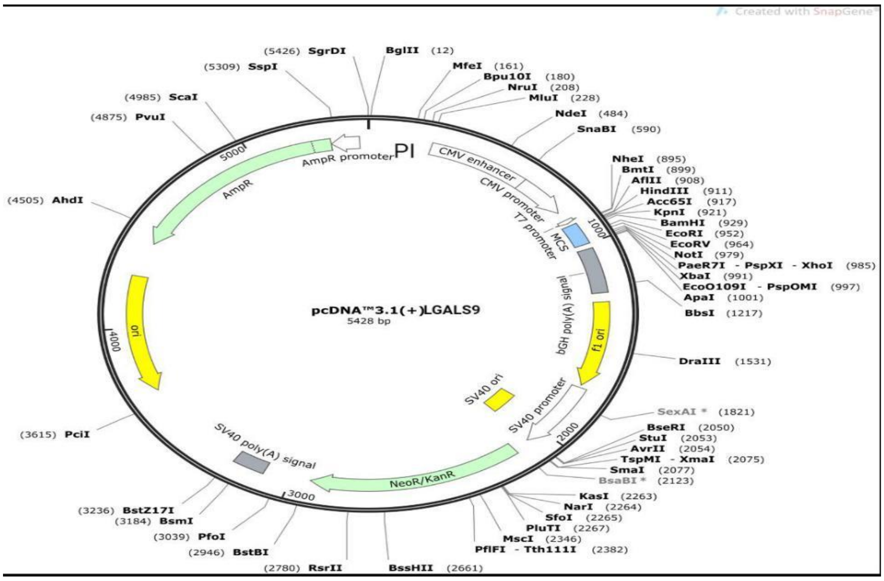

Supplementary Table S1

Table S1. Primers sequence used for miRNA and si-RNA

OLIGONUCLEOTIDE	SEQUENCE(5′-3′)
**bta-miR-24-3p mimics**	Sense -UGGCUCAGUUCAGCAGGAACAGTT-
	Antisense -GUUCCUGCUGAACUGAGCCAUUTT-
**Mimic Negative control**	Sense -UUCUCCGAACGUGUCACGUTT-
	Antisense -ACGUGACACGUUCGGAGAATT-
**bta-miR-24-3p inhibitors**	Sense -CUGUUCCUGCUGAACUGAGCCA-
**Inhibitor Negative control**	Sense- -CAGUACUUUUGUGUAGUACAA-
**Si-LGALS9**	Sense -GGAAGACACACAUGCCUUUTT-
	Antisense -AAAGGCAUGUGUGUCUUCCTT-
**Si-NC**	Sense -UUCUCCGAACGUGUCACGUTT-
	Antisense -ACGUGACACGUUCGGAGAATT-

Supplementary Table S2

Table S2. Primers used for quantitative reverse transcription PCR (RT-qPCR).

GENE	PRIMER SEQUENCE(5′-3′)	TM(oC)	SIZE(bp)
**IL-1β**	F -ACCAGCTCTACAACAAAAGA-	54	157

	R -TTGCACTTTACTGACTGCAC-		
**IL-6**	F -AGGCAGACTACTTCTGACCA-<br>R -TACTCCAGAAGACCAGCAGT	55	160
**IL-8**	F-CTGCAGTTCTGTCAAGGATG-<br>R-CAACCTTCTGCACCCACTTT-	55	160
**TNF*α***	F-CAGTCTCCTACCAGACCAAG-<br>R-CAGCATAGTCCAGGTAGTCC-	57	160
**LGALS9**	F-CTTTCATCACCACCATTCTG-<br>R-ATGTGGAACCTCTGAGCAC-	60	160
**TLR4**	F-AGACGACACATTTCAGGGCC-<br>R-CCAGGTTGGGCAGGTTAGAA-	60	160
**TRAF6**	F-GAGACAGGTTTCTTGTGACAAC-<br>R-TGGCAACCAAAAGTACTGAATG-	57	160
**IRAK4**	F-CAAGTGATGGAGATGACCTC-<br>R-TCTAGCAATAACTGAGGTTC-	57	160
**MyD88**	F-CGCGACGACGTGCTGATGGA-<br>R-TCGCTGGGGCAGTAGCAGAT-	60	160
**β-actin**	F-GACATCAAGGAGAAGCTCTG-<br>R-TGGAATTGAAGGTAGTTTCG-	60	120
